# Efficacy of blood purification for severe pancreatitis and acute respiratory distress syndrome

**DOI:** 10.1097/MD.0000000000017284

**Published:** 2019-09-20

**Authors:** Yi-hua Jin, Yang Liu

**Affiliations:** Department of Emergency, First Affiliated Hospital of Jiamusi University, Jiamusi, China.

**Keywords:** acute respiratory distress syndrome, blood purification, efficacy, randomized controlled trial, safety, severe pancreatitis

## Abstract

**Background:**

This study will assess the efficacy and safety of blood purification (BP) for severe pancreatitis (SP) and acute respiratory distress syndrome (ARDS).

**Methods:**

We will search the following electronic databases of Ovid MEDLINE, EMBASE, Web of Science, Cochrane Library, Scopus, Cumulative Index to Nursing and Allied Health Literature, the Allied and Complementary Medicine Database, Chinese Biomedical Literature Database, China National Knowledge Infrastructure, and WANGFANG from inception to the present without language restriction. A systematic review and data synthesis will be carried out of randomized controlled trials of BP for the treatment of patients with SP and ARDS. RevMan 5.3 software will be used for statistical analysis.

**Results:**

This systematic review will evaluate the efficacy and safety of BP for the treatment of patients with SP and ARDS. The primary outcome includes respiratory indexes, blood biochemical and inflammatory factors. The secondary outcomes consist of complications, sepsis, abdominal hemorrhage, renal failure, length of hospital stay, and mortality.

**Conclusion:**

This study will provide up-to-date evidence of BP for the treatment of patients with SP and ARDS.

**PROSPERO registration number:**

PROSPERO CRD42019139467.

## 1 Introduction

Severe pancreatitis (SP) is a very common gastrointestinal disorder in clinical practice.^[1–3]^ It often results from a release of high levels of inflammatory mediators in the pancreas,^[4,5]^ and is characterized by pancreatic digestion by its own enzymes and necrosis.^[4,5]^ Such disorder often involves multiple organ dysfunction syndromes, and leads to high mortality.^[6–9]^ Additionally, SP is often complicated acute respiratory distress syndrome (ARDS),^[10,11]^ and its associated mortality rate varies from 25% to 30%.^[10–13]^ Thus, early prevention and treatment for SP are essential to enhance prognosis and the quality of life in patients with SP and ARDS.^[14–16]^

Early blood purification (BP) is very crucial and important to clear inflammatory cytokines produced at inflamed sites.^[17–20]^ In addition, this treatment can also promote the recovery of immune function and stabilizes the internal environment.^[18–20]^ Although previous studies have reported the efficacy of BP for the treatment of patients with SP and ARDS,^[17–20]^ its efficacy is still inconclusive and is not supported by the study of systematic review. Therefore, this study will systematically assess its efficacy and safety of BP for the treatment of patients with SP and ARDS.

## 2 Methods

### 2.1 Study registration

This protocol has been registered online on the PROSPERO (CRD42019139467), and it has followed the guidelines of Preferred Reporting Items for Systematic Reviews and Meta-Analysis (PRISMA) Protocol statement.^[21]^

**Ethics and dissemination:** No research ethics approval is needed for this study, because no confidential patient data will be utilized in this systematic review. Its results will be published at a peer-reviewed journal or through conference presentations.

### 2.2 Study selection criteria

#### 2.2.1 Type of studies

We will only include randomized controlled trials (RCTs) of BP for the treatment of patients with SP and ARDS. All other studies except RCTs will be excluded.

#### 2.2.2 Type of interventions

Study reporting results of interventions involving BP alone for the treatment of patients with SP and ARDS will be included in the experimental group.

The control treatment will include any treatments, except BP.

#### 2.2.3 Type of population

We will include studies of patients with SP and ARDS regardless race, gender, and age.

### 2.3 Type of outcomes

The primary outcome includes respiratory indexes (PaO2, alveolar-arterial oxygen difference, respiratory rate, and related indexes), blood biochemical (creatinine, blood urea nitrogen, alanine aminotransferase, and lactate levels) and inflammatory (such as serum C-reactive protein, interleukin-6, and other related indexes).

The secondary outcomes consist of complications, sepsis, abdominal hemorrhage, renal failure, length of hospital stay, and mortality.

### 2.4 Search strategy

Ten electronic databases of Ovid MEDLINE, EMBASE, Web of Science, Cochrane Library, Scopus, Cumulative Index to Nursing and Allied Health Literature, Allied and Complementary Medicine Database, Chinese Biomedical Literature Database, China National Knowledge Infrastructure, and WANGFANG will be searched from inception to the present without language restriction. The full search strategy for Ovid MEDLINE is available in Table 1. Similar search strategy will also be adapted to other electronic databases.

**Table 1:**
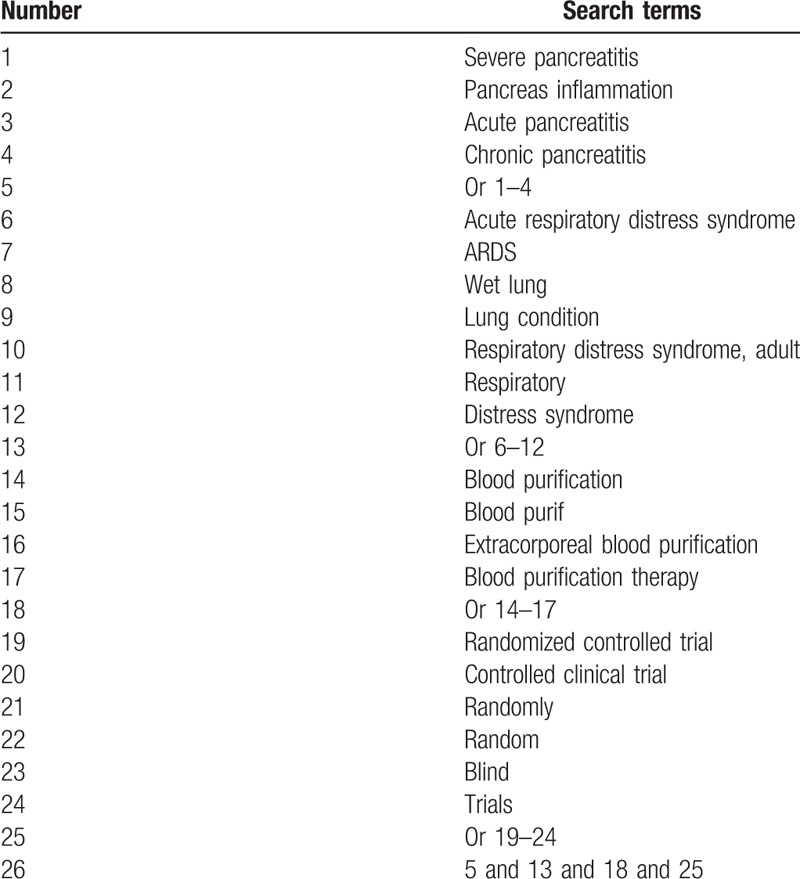
Search strategy for Ovid MEDLINE.

Additionally, grey literature will consist of unpublished conference proceedings or abstracts from relevant conferences, and reference lists of associated reviews.

### 2.5 Study selection process

**Figure 1:**
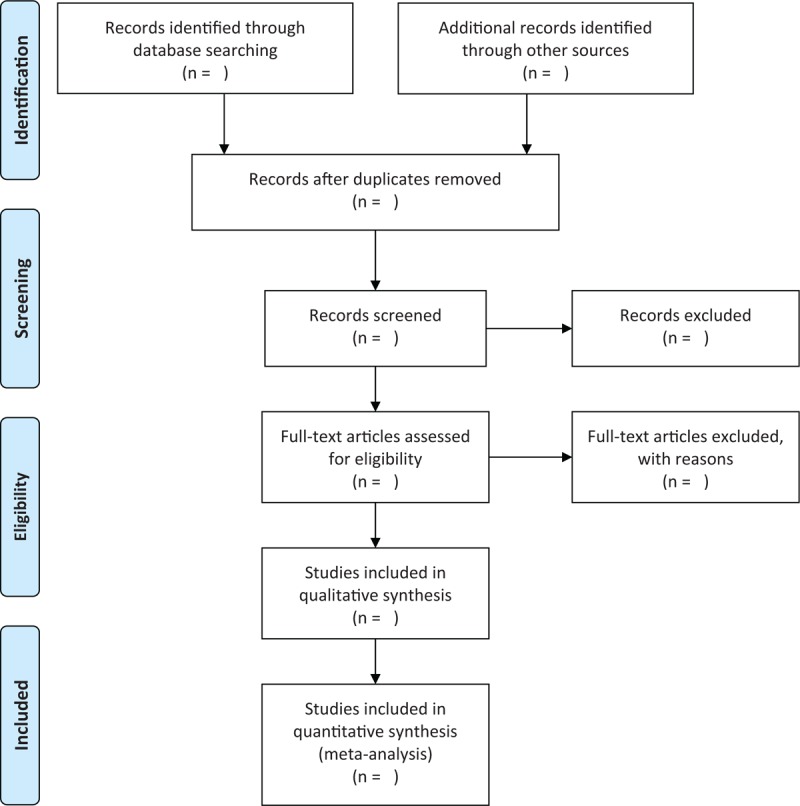
Flowchart of study selection.

Two independent researchers will carry out study selection according to the pre-designed inclusion and exclusion criteria. A third researcher will solve any conflicts between two researchers through discussion. We will scan all titles and abstracts from all searched records, and we will exclude all irrelevant records. After that, all remaining studies will be read by full texts. The flowchart of study selection is exerted in Figure 1.

### 2.6 Data extraction and management

Two independent researchers will extract all relevant data based on the predefined data extraction form. Any conflicts regarding data extraction between two researchers will be solved by a third researcher via discussion. Such form contains study setting, study population, patient characteristics, diagnosis criteria, inclusion and exclusion criteria, study methods, treatment details, and outcome measurements.

### 2.7 Risk of bias assessment

Cochrane Risk of Bias Tool will be applied for risk of bias assessment for each eligible study by two independent researchers. Any discrepancies or unusual patterns will be solved by a third researcher via discussion. This tool comprises of 7 items, and each item will be assessed as 3 levels: low, unclear, and high risk of bias.

### 2.8 Treatment effect measurement

In this study, the continuous values will be calculated as mean difference or standardized mean difference and 95% confidence intervals. The dichotomous values will be calculated as risk ratio with 95% confidence intervals.

### 2.9 Missing data dealing with

We will contact primary authors from original papers if there is missing data in the eligible studies. Only available data will be analyzed if we can not require those data.

### 2.10 Heterogeneity identification

In this study, *I*^2^ test will be used to identify the heterogeneity. If the heterogeneity is less than 50%, it will be regarded as minor, and a fixed-effect model will be used. If the heterogeneity is more than 50%, it will be considered as significant and a random-effect model will be applied.

### 2.11 Data synthesis

If minor heterogeneity is identified (*I*^2^ ≤ 50%), we will carry out meta-analysis when it is possible. Otherwise, if significant heterogeneity is found (*I*^2^ > 50%), we will perform subgroup analysis. If there is still significant heterogeneity after subgroup analysis, we will not conduct meta-analysis, but will only describe outcome results as narrative summary.

### 2.12 Subgroup analysis

We will carry out subgroup analysis to identify possible reasons that may cause substantial heterogeneity based on the differences of treatments, controls, and outcome assessments.

### 2.13 Sensitivity analysis

We will perform sensitivity analysis to explore the robustness and stability of pooled outcome results by removing low quality studies.

### 2.14 Publication bias

We will conduct funnel plot,^[22]^ Egger's regression and Begger's tests to check if there are any publication biases among eligible studies.^[23]^

## 3 Discussion

Previously, no study has explored the efficacy and safety of BP for the treatment of SP and ARDS, although a variety of studies have reported its benefits for patients with SP and ARDS. This study will apply rigorous methodology to examine studies reporting the outcomes of BP for the treatment of patients with SP and ARDS. The results of study will inform our understanding of the efficacy of BP in treating SP and ARDS outcomes. The results may also provide helpful evidence for clinician and health policy-makers for treating SP and ARDS.

## Acknowledgments

This study is supported by the Heilongjiang Provincial Health and Family Planning Commission Research Project (NO.2018–084). Provider did not involve all sections of this study.

## Author contributions

**Conceptualization:** Yi-hua Jin, Yang Liu.

**Data curation:** Yi-hua Jin, Yang Liu.

**Formal analysis:** Yi-hua Jin, Yang Liu.

**Funding acquisition:** Yi-hua Jin.

**Investigation:** Yi-hua Jin.

**Methodology:** Yang Liu.

**Project administration:** Yi-hua Jin.

**Resources:** Yang Liu.

**Software:** Yang Liu.

**Supervision:** Yi-hua Jin.

**Validation:** Yi-hua Jin, Yang Liu.

**Visualization:** Yi-hua Jin, Yang Liu.

**Writing – original draft:** Yi-hua Jin, Yang Liu.

**Writing – review & editing:** Yi-hua Jin, Yang Liu.
